# Antihypertensive Effects of IGTGIPGIW Peptide Purified from *Hippocampus abdominalis*: p-eNOS and p-AKT Stimulation in EA.hy926 Cells and Lowering of Blood Pressure in SHR Model

**DOI:** 10.3390/md20060354

**Published:** 2022-05-26

**Authors:** Hyo-Geun Lee, Hyun-Soo Kim, Hyesuck An, Kyunghwa Baek, Jeong Min Lee, Mi-Jin Yim, Seok-Chun Ko, Ji-Yul Kim, Gun-Woo Oh, Jun-Geon Je, Dae-Sung Lee, You-Jin Jeon

**Affiliations:** 1 Department of Marine Life Science, Jeju National University, Jeju 63243, Korea; hyogeunlee92@gmail.com (H.-G.L.); wpwnsrjs@gmail.com (J.-G.J.); 2 National Marine Biodiversity Institute of Korea, 75, Jangsan-ro 101-gil, Janghang-eup, Seocheon 33362, Korea; gustn783@mabik.re.kr (H.-S.K.); mgran@mabik.re.kr (H.A.); kyunghwabaek@mabik.re.kr (K.B.); lshjm@mabik.re.kr (J.M.L.); mjyim@mabik.re.kr (M.-J.Y.); seokchunk@mabik.re.kr (S.-C.K.); jiyul2224@mabik.re.kr (J.-Y.K.); ogwchobo@mabik.re.kr (G.-W.O.)

**Keywords:** *Hippocampus abdominalis*, EA.hy926, peptide, antihypertensive activity

## Abstract

The aim of this study was to assess the potential hypertensive effects of the IGTGIPGIW peptide purified from *Hippocampus abdominalis* alcalase hydrolysate (HA) for application in the functional food industry. We investigated the antihypertensive effects of IGTGIPGIW in vitro by assessing nitric oxide production in EA.hy926 endothelial cells, which is a major factor affecting vasorelaxation. The potential vasorelaxation effect was evaluated using 4-amino-5-methylamino-2′,7′-difluorofluorescein diacetate, a fluorescent stain. IGTGIPGIW significantly increased the expression of endothelial-derived relaxing factors, including endothelial nitric oxide synthase and protein kinase B, in EA.hy926 cells. Furthermore, oral administration of IGTGIPGIW significantly lowered the systolic blood pressure (183.60 ± 1.34 mmHg) and rapidly recovered the diastolic blood pressure (143.50 ± 5.55 mmHg) in the spontaneously hypertensive rat model in vivo. Our results demonstrate the antihypertensive activity of the IGTGIPGIW peptide purified from *H. abdominalis* and indicate its suitability for application in the functional food industry.

## 1. Introduction

Hypertension (HTN) is a chronic disease prevalent worldwide [1,2,3]. According to the World Health Organization, more than 1 billion people have suffered from HTN since 2019 [4]. In their study on the global epidemiology of HTN, Mill et al. reported that 1.39 billion out of the adult population suffered from HTN, and the prevalence of adult HTN was higher in low- and middle-income countries than in high-income countries [5]. Presently, childhood HTN, which represents another public health concern, is recognized as an important parameter of cardiovascular disease in adults [6,7,8]. Therefore, the proper management of childhood HTN is important to prevent both cardiovascular diseases and HTN during adulthood [9]. HTN is characterized by high arterial blood pressure and is associated with a high risk of cardiovascular diseases, including coronary disease, valvular disease, ventricular hypertrophy, arrhythmias, stroke, and retinal vein occlusions [10]. It has been shown that HTN is often accompanied by metabolic comorbidities, including diabetes and obesity [11,12,13], and is frequently observed in patients with diabetes mellitus [14]. Some studies have reported the correlation between HTN and chronic kidney disease (CKD) resulting in liver cirrhosis [15,16], and Steven et al. (1993) reported an association between hypertension and asthma during pregnancy [17]. Earlier reports showed that most marine-derived peptides exhibit biological activities and are particularly strong in their antihypertensive activity [18,19,20]. Of the different source species, marine fish have been assessed to study the effects of hydrolysates derived from them on patients with hypertension [21,22,23]. Some studies have focused on the potential antihypertensive effects of fish byproducts, such as fish intestines, bones, or collagen from fish skin [24,25,26]. Soudabeh et al. investigated the potential inhibitory effect of peptides present in codfish blood and water remaining after cooking sardines on the angiotensin-converting enzyme (ACE) activity [27]. Ecofriendly research has increased the value of previously “useless” byproducts of the fish processing industry. Therefore, we evaluated the potential antihypertensive effects of a peptide purified from alcalase hydrolysates of *Hippocampus abdominalis* (*H. abdominalis*). With growing recognition of the bioactivities of this peptide, many researchers have focused on the development of new peptides from land or marine animals. Recently, many publications have reported on the importance of marine-derived peptides, and Shukla et al. (2016) reviewed the unique structures of marine-derived peptides and insisted that this novel type of peptide has beneficial functions [28]. In the present study, we examined the antihypertensive effect of IGTGIPGIW, a peptide purified from *H. abdominalis*, on EA.hy926 endothelial cells. HA-Ⅲ-b presented excellent ACE inhibitory activity in our previous study [29]. The present study is an extended investigation of the potential antihypertensive effects of IGTGIPGIW in EA.hy926 cells and in a spontaneously hypertensive rat (SHR) model. A 4-Amino-5-methylamino-2′,7′-difluorofluorescein diacetate (DAF-FM-DA) assay was used to detect nitric oxide (NO) generation in EA.hy926 cells treated with IGTGIPGIW. NO is an important vasorelaxation factor in human blood vessel endothelial cells. In addition, we evaluated the effects of IGTGIPGIW on phospho-endothelial nitric oxide synthase (p-eNOS) and phospho-protein kinase B (p-AKT) by a Western blot assay. Furthermore, we measured the systolic blood pressure (SBP) and diastolic blood pressure (DBP) in an IGTGIPGIW-treated SHR model.

## 2. Results

### 2.1. Non-Cytotoxicity of IGTGIPGIW in EA.hy926 Cells

**Figure 1:**
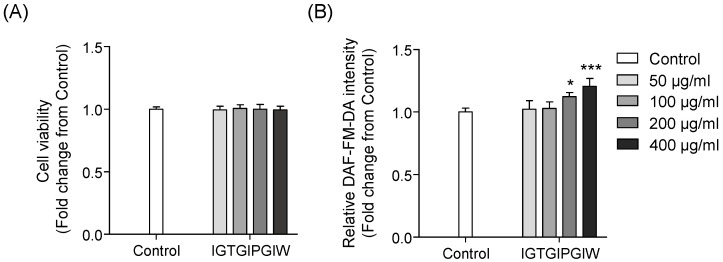
(**A**) Cell viability and (**B**) DAF-FM-DA intensity in EA.hy926 cells. Cell viability was determined with MTT assay and DAF-FM-DA staining was performed to evaluate the NO production. All data were presented as fold changes from the control and the results were expressed as the mean ± SD of three separate experiments. Significant differences were identified at * *p* < 0.05, *** *p* < 0.001 compared to the control group.

The cytotoxic effects of IGTGIPGIW were determined by the MTT assay after exposure to IGTGIPGIW for 24 h and expressed as a graph following the relative crystal violet production in viable cells. As shown in Figure 1, no significant difference was detected in the cell viability of the control and IGTGIPGIW-treated groups. The results indicate that the purified IGTGIPGIW peptide and its selected treatment range did not exert any cytotoxic effects on EA.hy926 cells.

### 2.2. Effects of IGTGIPGIW on NO Generation in EA.hy926 Cells

**Figure 2:**
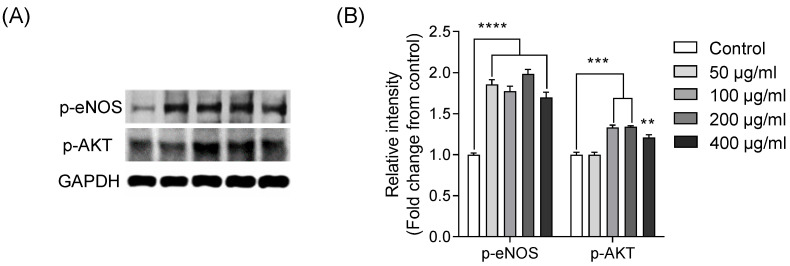
Peptide IGTGIPGIW stimulates the p-eNOS and p-AKT expression in EA.hy926 cells. The protein expressions of p-eNOS and p-AKT assessed by Western blot analysis (**A**) Western blot bands of phosphorylated and phosphorylated eNOS. (**B**) Quantification of p-eNOS and p-AKT protein expressions in EA.hy926 cells treated peptide IGTGIPGIW. All the data were presented as fold changes from the control and expressed as the mean ± SD of three separate experiments. Significant differences were identified at ** *p* < 0.01, *** *p* < 0.001, **** *p* < 0.0001 compared to the control group.

Endothelial NO is an important biomarker of vasoconstriction in blood vessels [30]. Therefore, we tested the effects of IGTGIPGIW from *H. abdominalis* on NO production in EA.hy926 cells using the DAF-FM-DA fluorescent dye, which specifically detects NO. As shown in Figure 2, the relative DAF-FM-DA intensity indicated significantly higher NO production in groups treated with high concentrations of IGTGIPGIW (200 and 400 μg/mL) than in the control group. The results clearly demonstrate that IGTGIPGIW increased NO generation in EA.hy926 cells, indicating its significance in intracellular NO generation.

### 2.3. Vasorelaxation Mediated via p-AKT and p-eNOS Pathways in EA.hy926 Cells Treated with IGTGIPGIW

As mentioned previously, IGTGIPGIW can regulate NO generation in EA.hy926 cells. Therefore, we explored the antihypertensive effect of IGTGIPGIW on the eNOS and AKT pathways, which represent major vasorelaxation pathways. Moreover, the activation of eNOS/AKT mechanisms can induce vasorelaxation [31]. The upstream regulation of p-eNOS and p-AKT via signaling molecules was observed in IGTGIPGIW-treated EA.hy926 cells. Collectively, these results indicate that the purified IGTGIPGIW peptide could promote p-eNOS and p-AKT expression in EA.hy926 cells.

### 2.4. Effects of IGTGIPGIW on SBP and DBP of SHRs

**Figure 3:**
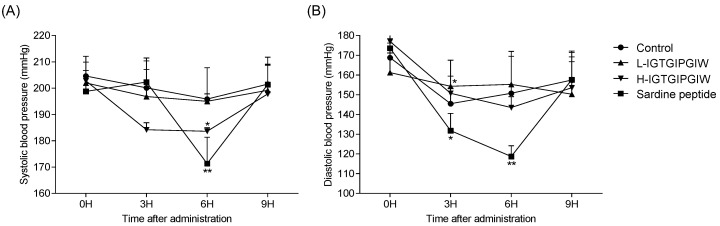
Effects of peptide IGTGIPGIW on systolic and diastolic blood pressure in spontaneous hypertensive rat. (**A**) Time-interval systolic and (**B**) diastolic blood pressure changes in SHR-treated peptide IGTGIPGIW. (●) Control (water); (▲) L-IGTGIPGIW (50 mg/kg); (▼) H-IGTGIPGIW (100 mg/kg); (■) Sardine peptide (50 mg/kg). Values were expressed as the mean ± SD (*n* = 5/group). Significant differences were identified at * *p* < 0.05, ** *p* < 0.01 compared to the control group.

Figure 3 shows the effects of IGTGIPGIW on the SBP of SHRs. The short-term antihypertensive efficacy of IGTGIPGIW on SBP and DBP was observed for 12 h using the CODA Tail-Cuff Blood Pressure System (KENT Scientific Co., Torrington, CT, USA). The experimental SHRs were randomly divided into four groups and orally administered with low (50 mg/kg) and high (100 mg/kg) concentrations of IGTGIPGIW. Initially, high SBP (202.32 ± 5.86 mmHg) was maintained in all groups. A slight reduction in SBP was observed in the 50-mg/kg IGTGIPGIW group compared with that of the control group, albeit without significance. However, H-IGTGIPGIW (100 mg/kg) significantly lowered SBP after treatment for 3 and 6 h. After 3 and 6 h of H-IGTGIPGIW treatment, the SBP decreased to 184.20 ± 2.68 and 183.60 ± 1.34 mmHg, respectively. Similarly, the initial DBP of the SHRs averaged 167.55 ± 10.47 mmHg and was not significantly different between the control and IGTGIPGIW-treated groups. However, the IGTGIPGIW peptide significantly decreased the DBP to 131.83 ± 8.75 mmHg and 118.71 ± 5.41 mmHg after treatment for 3 and 6 h, respectively. These results indicate that the purified IGTGIPGIW peptide could control high blood pressure in the SHR model by downregulating SBP.

### 2.5. Chromatographic Analysis of the Purified IGTGIPGIW Peptide

**Figure 4:**
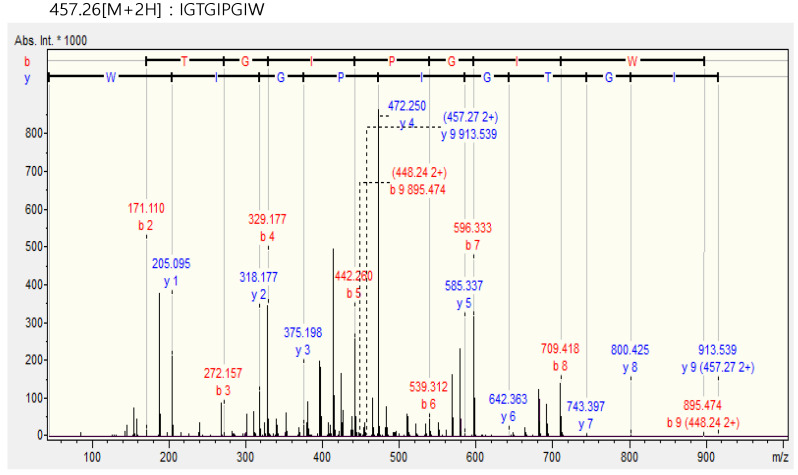
LC-MS/MS measurement and amino acid sequencing of peptide IGTGIPGIW isolated from *Hippocampus abdominalis* (*H. abdominalis*).

IGTGIPGIW purified from *H. abdominalis* was analyzed by liquid chromatography-tandem mass spectrometry (LC-MS/MS). The molecular mass and amino sequences determined are presented in Figure 4. The LC-MS/MS spectrum of IGTGIPGIW showed peaks at 913.539, 800.425, 743.397, 642.363, 585.337, 457.27, 472.250, 375.198, 318.177, and 205.095 *m*/*z*. The mass peaks at 913.539–800.425 *m*/*z* (isoleucine; Ile), 800.425–743.397 *m*/*z* (glycine; Gly), 743.397–642.363 *m*/*z* (threonine; Thr), 642.363–585.337 *m*/*z* (Gly), 585.337–457.27 *m*/*z* (Ile), 457.27–472.250 *m*/*z* (proline; Pro), 472.250–375.198 *m*/*z* (Gly), 375.198–318.177 *m*/*z* (Ile), and 318.177–205.095 *m*/*z* (tryptophan; Trp) indicated a polypeptide fragment of Ile-Gly-Thr-Gly-Ile-Pro-Gly-Ile-Trp (IGTGIPGIW). The molecular mass of purified IGTGIPGIW was calculated as 914.52 Da.

## 3. Discussion

HTN is a chronic medical condition characterized by persistently elevated arterial blood pressure. It has been recognized as a global epidemiologic disease and is prevalent in 31.1% (1.39 billion) of the global adult population. HTN is a major risk factor for cardiovascular diseases, including heart failure, heart valve diseases, atrial fibrillation, aortic syndrome, and coronary heart disease, leading to complications in patients [32]. Borzecki et al. reported that HTN complications or comorbidities include cardiovascular diseases, asthma, diabetes, hyperlipidemia, and renal disease [33]. Fang et al. suggested that patients with HTN and diabetes mellitus are more vulnerable to the novel coronavirus disease [34]. Therefore, the prevention and control of HTN are important to prevent severe HTN-induced comorbidities. However, the control of HTN-induced comorbidities is more difficult in patients with severe secondary HTN. According to the 2017 American College of Cardiology/American Heart Association guidelines, complications caused by HTN-associated comorbidities are often reported in patients with HTN [35].

The seahorse is a teleost marine fish belonging to the family Syngnathidae. Most Hippocampus spp. occur in the Indo-Pacific Ocean and New Zealand. To date, 47–57 seahorse species belonging to the genus Hippocampus have been identified [36]. Moreover, the seahorse is a valuable marine animal with global applications in traditional medicine [37]. It is a source of several steroids, minerals, peptides, and fatty acids, which exhibit various pharmacological activities, including anti-tumor, anti-aging, anti-fatigue, anti-thrombus, anti-arthritis, anti-oxidative, and anti-prostatic activities [38]. A recent polypeptide isolated from *H. abdominalis* exhibited excellent antioxidant and anti-fatigue activities in vivo [39]. Moreover, the ethanol extract showed an anti-melanogenesis effect in B16F10 cells in vitro [40]. Seahorses are known to have nutritional and health benefits and have been commercially exploited for decades in many countries [41,42,43].

In the present study, we evaluated the potential antihypertensive effects of IGTGIPGIW from *H. abdominalis* on EA.hy926 cells in vitro. The peptide was shown to be non-cytotoxic and to significantly increase the NO levels in vitro. NO is a major molecule regulating vasorelaxation and is known to affect blood pressure. Several studies have reported an association between blood pressure and gaseous NO synthesis [44,45]. Moreover, NO can control blood pressure and maintain tension in blood vessels. This is important in patients with cardiovascular diseases and CKD who have blood pressure anomalies [46,47]. Furthermore, the Western blot analysis revealed that the IGTGIPGIW peptide significantly increased the levels of p-eNOS and p-AKT in EA.hy926 cells. Thus, the IGTGIPGIW peptide of *H. abdominalis* could regulate NO production, resulting in vascular relaxation via the upregulation of eNOS and AKT expression in EA.hy926 cells. These results indicated the strong antihypertensive effect of IGTGIPGIW via the relaxation of endothelial-dependent mechanisms. A short-course oral administration of a single dose of H-IGTGIPGIW peptide effectively lowered SBP and DBP in the animal model in vivo. These results corresponded with the findings of Rahmdel et al. on the antihypertensive effect of flounder fish peptide [48].

In conclusion, the present study demonstrated that the IGTGIPGIW peptide purified from *H. abdominalis* is a potent NO promoter in EA.hy926 endothelial cells. Furthermore, IGTGIPGIW significantly lowered the SBP and DBP in the SHR model. We hypothesized that the antihypertensive activity was mediated by vasorelaxation-associated eNOS and AKT signaling pathways. These results indicated that the alcalase-assisted hydrolysate from *Hippocampus abdominalis* containing bioactive IGTGIPGIW, antihypertensive peptide, and the antihypertensive peptide containing seahorse hydrolysate can be utilized as raw materials in the food and functional food industries. Further, this study provides a practical application for alcalase-assisted hydrolysate from *Hippocampus abdominalis*.

## 4. Materials and Methods

### 4.1. Materials and Chemicals

*Hippocampus abdominalis* (*H. abdominalis*) were obtained from the Centre for Ornamental, Reef, and Aquarium at Jeju Island, South Korea. Commercial food-grade alcalase (EC 3.4.21.62) was purchased from Novozyme (Bagsvaerd, Copenhagen, Denmark). Cells were cultured in Dulbecco’s modified Eagle’s medium (DMEM; catalog number 12430-054), fetal bovine serum (FBS; catalog number 16000-044), penicillin/streptomycin (P/S; catalog number 15140-122), and trypsin-EDTA (catalog number 15400-054) obtained from GIBCO-BRL (Grand Island, NY, USA). Thiazolyl blue tetrazolium bromide (MTT; catalog number 0793) and dimethyl sulfoxide (DMSO; catalog number 0231) were purchased from VWR Life Science (Lutterworth, UK). Cell culture plates were purchased from SPL Life Sciences (Pocheon, South Korea). The primary antibodies, p-eNOS (catalog number 9571S) and p-AKT (catalog number 9271), were purchased from Cell Signaling Technology (Bedford, MA, USA). GAPDH (catalog number sc-66163) was purchased from Santa Cruz Biotechnology (Santa Cruz, CA, USA). Horseradish peroxidase (HRP)-conjugated secondary antibodies (catalog number sc-516102) were purchased from Santa Cruz Biotechnology (Santa Cruz, CA, USA). The ECLTM anti-rabbit IgG HRP-linked whole secondary antibodies were purchased from GE Healthcare (NA934V, GE Health, Buckinghamshire, UK).

### 4.2. Purification of IGTGIPGIW from Alcalase HydrolysaSte of H. abdominalis

Peptides were extracted and purified from *Hippocampus abdominalis* alcalase hydrolysate (HA) following the procedures reported by Kim et al. In brief, dried seahorse (5 g) was mixed with 100 mL of distilled water containing 10 mg of alcalase enzyme and then extracted under optimal conditions (pH 4.5, 37 °C) for 24 h. After hydrolyzation for 24 h, alcalase-assisted hydrolysates were centrifuged (12,902× *g* rpm, 4 °C, 15 min) and the supernatant was filtered followed by heat inactivation (105 °C, 10 min). The hydrolysates were then subjected to separating membranes and the hydrolysates were then fractionated using an ultrafiltration system (Labscale Tangential Flow Filtration System, Millipore, Merck KGaA, Darmstadt, Germany) equipped with molecular weight cutoff (MWCO) membranes. The hydrolysates were fractionated using two MWCO membranes in decreasing sizes (10 kDa and 5 kDa). Three fractions (>10 kDa, HA-Ι; 5–10 kDa, HA-ΙΙ; <5 kDa, HA-ΙΙΙ) were obtained. Of the fractions collected, the HA-III (<5 kDa) fraction was loaded onto a Sephadex G10 open column to separate the active peptide. Ultimately, four peptide fractions (HA-ΙΙΙ-a, HA-ΙΙΙ-b, HA-ΙΙΙ-c, and HA-ΙΙΙ-d) were obtained, and IGTGIPGIW, the potential antihypertensive peptide of interest, was isolated from HA-ΙΙΙ-b.

### 4.3. Cell Culture and Cytotoxicity Analysis

EA.hy926 cells were purchased from the American Type Culture Collection (Rockville, MD, USA) and were cultured in DMEM (10% FBS, 1% P/S). The cytotoxic effect of IGTGIPGIW was analyzed by the MTT assay. Briefly, EA.hy926 cells were seeded in 96-well plates and incubated at 37 °C, in a 5% CO_2_ maintained environment for 24 h. Then, they were treated with different concentrations of IGTGIPGIW (50–400 μg/mL) and incubated (37 °C, 5% CO_2_) for 24 h. All treatments consisted of three replicates. After 24 h, 50 μL of the MTT stock solution (2 mg/mL) was added to each well, followed by incubation for 2–3 h. The generated formazan crystals were dissolved in DMSO and detected using a microplate reader (Synergy HT Multi-Detection microplate reader, Bio-Tek, Winooski, VT, USA). Cell viability was expressed with respect to the control (non-treated sample).

### 4.4. Amino Acid Sequence Analysis

The amino acid sequence and molecular weight were analyzed using an HPLC system (Waters, Milford, MA, USA) connected to a Q-TOF mass spectrometer (Micromass, Altrincham, UK) under electrospray ionization. The purified IGTGIPGIW peptide was loaded onto a C-18 ODS column (4.6 mm × 150 mm). The conditions for the mass spectrometer were as follows: flow rate, 100 μL/min; scan range, 50–2000 *m*/*z*; source temperature, 180 °C. Chromatography and mass spectrometry were performed following the procedures reported by Kim et al. [29].

### 4.5. Detection of NO Production in Endothelial Cells

Intracellular NO production was determined using DAF-FM-DA, a fluorometric NO indicator. EA.hy926 cells were seeded in 96-well plates at a concentration of 1 × 10^5^ cells/mL and incubated at 37 °C under 5% CO_2_ humidified conditions for 24 h. Once the plated cells reached 100% confluence, 10 μL of DAF-FM-DA (10 mM) was added and the culture was incubated for 30 min. The cell culture medium was then removed and washed twice with 100 μL of 1X phosphate buffer saline (PBS). Then, all wells were filled with 100 μL of 1X PBS solution, and the intensity of DAF-FM-DA was measured using a microplate reader (Synergy HT Multi-Detection microplate reader, Bio-Tek, Winooski, VT, USA) at excitation and emission wavelengths of 485 nm and 520 nm, respectively.

### 4.6. Western Blot Assay

The harvested EA.hy926 cells were homogenized in lysis buffer (20 mM Tris, 10 mM Na_4_P_2_O_7_, 2 mM Na_3_VO_4_, 1 mM PMSF, 100 mM NaF, 10 mg/mL leupeptin, 5 mM EDTA, 10 mg/mL aprotinin, and 1% NP-40) for 1 h on ice. The cell lysates were clarified by centrifugation at 4 °C and at 12,000 rpm for 20 min. The supernatant was collected, and the protein content was measured using a bicinchoninic acid protein quantification kit. Then, the protein concentration was normalized to 10 μg/mL, and the cell lysates were mixed with dithiothreitol and 4X loading buffer. Subsequently, an equal amount of protein was electrophoresed on a sodium dodecyl sulfate-polyacrylamide gel and transferred to a nitrocellulose membrane, which was incubated overnight at 4 °C with antihypertensive-specific primary antibodies (1:1000) (p-eNOS and AKT). The membranes were washed with 1X TBST solution. Then, the membranes were incubated with secondary antibodies (1:3000) for 2–3 h at room temperature. The target bands were detected using the Fusion Solo imaging system (Vilber Lourmat, France) with an enhanced chemiluminescence Western blot detection kit (SuperSignal West Femto Maximum Sensitivity Substrate, Thermo Scientific, Pittsburgh, PA, USA). These protein bands were then quantified using ImageJ software.

### 4.7. Experimental Animals

Twenty SHRs were purchased from the Jung Ang Lab Animal Inc. (Seoul, South Korea). The Institutional Animal Care and Use Committee of Jeju National University approved the use of SHRs (4–5 weeks old, female) for the experiments. The rats were housed under optimal environmental conditions (20–22 °C, 55% humidity) and a 12-h light:dark photoperiod. After 1 week of environmental acclimation, the animals were divided into five groups (*n* = 5) and subjected to different treatments orally. Control group was administered the same volume of water; a group was treated with commercial sardine peptide (SP, 50 mg/kg); and groups were treated with L-IGTGIPGIW (50 mg/kg), H-IGTGIPGIW (100 mg/kg). All experiments were performed following the experimental animal guidelines of the Animal Care Center of Jeju National University (2017-0017).

### 4.8. Measurement of SBP and DBP

To assess the antihypertensive effect of the peptide purified from HA, the SBP and DBP of SHRs (7–8 weeks, 250~300 g body weight) were monitored using a tail-cuff volume pressure system (CODA, Kent scientific, Torrington, CT, USA). SBP and DBP were measured between 15:00 and 03:00 at three-hour intervals for a total of 12 h. Three independent measurements were averaged and presented as a graph.

### 4.9. Statistical Analyses

All experiments were performed in triplicate. Data were analyzed using GraphPad Prism version 6.01 and Microsoft Excel 2016. Results were expressed as means ± standard deviations and analyzed using one-way analysis of variance. Statistical significance was established at * *p* < 0.05, ** *p* < 0.01, and *** *p* < 0.001, and **** *p* < 0.0001 compared with the control.

## Data Availability

Not applicable.
